# Editorial: Regeneration and Brain Repair

**DOI:** 10.3389/fncel.2021.687992

**Published:** 2021-05-20

**Authors:** Daniella Rylander Ottosson, Sofia Grade, Andreas Heuer

**Affiliations:** ^1^Laboratory of Regenerative Neurophysiology, Department of Experimental Medical Sciences, Lund Stem Cell Centre, Lund University, Lund, Sweden; ^2^Institute of Molecular Biotechnology of the Austrian Academy of Sciences (IMBA), Vienna Biocenter (VBC), Vienna, Austria; ^3^Behavioural Neuroscience Laboratory, Department of Experimental Medical Sciences, Lund University, Lund, Sweden

**Keywords:** neurodegeneration, brain injury, brain repair, neuroprotection, neuronal reprogramming, cell therapy, stem cells

Brain disorders such as Parkinson's disease (PD), Alzheimer's disease, Multiple sclerosis, Huntington's disease (HD), and Stroke currently lack effective treatments and represent major healthcare challenges. With the aim to develop strategies for brain repair, the field of neural regeneration is constantly exploring exciting new and refined strategies. The current Research Topic has gathered new evidence and compiled past and recent efforts to achieve this goal. Altogether, four main subtopics are showcased (1) neuronal replacement strategies, mainly through cell transplantation, (2) neuroprotective approaches, (3) studies into the disease pathology to efficiently design the former strategies, and (4) adult neurogenesis as a valuable lens to neuronal development in an adult brain.

In contrast to many other mammalian organs, the brain lacks regenerative capacity (with some exceptions) and therefore, neurons can only be restored through an exogenous route, e.g., via cell transplantation. The past years have witnessed impressive developments in obtaining novel cell sources for cell transplantation ranging from the patients' own cells through reprogramming of skin fibroblasts to the generation of chimeric animals for xenotransplantation. The interest and efforts laid in this field are enormous and have led to the fascinating development of hESC (human embryonic stem cell)- and iPSC (induced pluripotent stem cell)-derived neuronal subtypes of clinical relevance. Indeed, human PSC-derived dopaminergic neurons for PD are now in clinical trials and similar studies using human PSC-derived striatal neurons in patients of HD are likely to follow. Further progress in this field will critically depend on a close interaction between experimental and clinical research. In the future, cell replacement strategies might also involve *in situ* cell type conversion e.g., via forced expression of neurogenic transcription factors in non-neuronal cells. This so-called neuronal reprogramming has been evolving at a fast pace and holds great potential since it relies on patients' own cells.

This Research Topic includes two unique reviews in the field of cell therapy for PD and HD. One in the shape of a 40-year perspective piece on circuitry repair in basal ganglia, presenting an overview of past and current efforts to restore neurotransmission or damaged connectivity in the adult mammalian brain. Here, pioneers in the field of cell replacement therapy using primary fetal tissue (Björklund) and hESCs (Parmar) cover the historical development of the field from its beginnings in the 1970s to those of today using alternative cell sources as donor tissue. The second review by Osborn et al. describes cell therapy for PD with a focus on donor cell types, engaging into a revision of advantages and drawbacks of autologous cells, and careful considerations on costs and benefits.

Despite great progress, there are still many efforts needed to validate that the lab-grown cells are *en pair* with the gold-standard primary tissue from fetal brain as well as to elucidate the exact mechanism of repair in a given paradigm. A new era of brain repair has arrived, where advances in single cell profiling help define cell identities and lineages progression, going hand-in-hand with rapidly expanding methods for *in vivo* reprogramming. On this theme, the review from the Brüstle laboratory covered major achievements and future prospects on transcription factor-guided differentiation and forward programming of PSCs (Flitsc et al.). The authors beautifully cover the most representative progress to derive clinically relevant specialized neuronal subtypes as well as glial cells and highlight the remaining challenges. With similar motivation, the Takahashi lab used a newly identified cell surface marker of corticospinal motor neurons progenitor cells L1CAM to enrich the donor cell suspension obtained from fetal tissue, into the cell type of interest for transplantation into rodents (Samata et al.). They report enhanced survival and ability to extend axonal projections in the corticospinal tract using L1CAM+ sorted fetal grafts cells as compared with non-sorted, reiterating the need for an identity match also in models of acute brain injury.

Besides cell transplantation or local reprogramming strategies, brain repair approaches also include disease-modifying strategies designed to retard the ongoing degeneration by neuroprotection, ultimately slowing down disease progression. On this theme, leucine-rich repeat kinase 2 (LRRK2) appears as a promising target to counteract multiple pathogenic processes underlying PD development as described by Calabresi and co-authors in the appraisal from Mancini et al. LRRK2 mutations are responsible for the majority of inherited familial PD cases, can also be found in sporadic PD, and might underlie early pathological phenomena. Along the same lines, Manfredsson et al. share an opinion article that assembles the points of discussion on glial derived-neurotrophic factor (GDNF) delivery in PD raised at the American Society of Neural Therapy and Repair and recommendations calling for improved preclinical models and methods of GDNF delivery, early diagnostics, and clinical trial design.

Furthermore, the present Research Topic includes contributions that provide fundamental insights into the biology of brain pathology. Prodromidou and Matsas illuminate the emergent role of miRNAs as master regulators of gene expression that rewire transcriptional landscapes during human brain development and neurological disease, a finding that instigated their use in therapeutics. From gene expression to cellular processes and extracellular matrix (ECM) alterations, a hybrid article from the Götz lab provides original results of proteome profiling after traumatic brain injury, framed into an elegant review of the field of glial scar biology, with a focus on ECM composition and wound healing (Kjell and Götz). A recurrent theme on various contributions was that not only disease mechanisms need to be better understood but also disease models need to be improved. Ermine et al. describe a model of endothelin-induced cortical ischemia in rats with a temporal progression of the behavioral deficits and of atrophy that better resemble those in stroke patients.

This collection also highlights research on adult neurogenesis that constitutes unarguably a hot topic in neurosciences and with implications for repair. In a review from Petrik and Encinas, we can follow the debate and controversy on the existence of adult neurogenesis in the human brain and its similarity to rodent neurogenesis. This review brings up the issues of technical criteria to identify adult neurogenesis in humans and entertains considerations about the temporal differences in neurogenesis decline in rodents vs. humans and the need to re-evaluate the existence of human neurogenesis out of the hippocampal niche. Given the current landscape on this topic we can certainly expect more studies to be added in the future and the discussion to continue. Indeed, the field of neural regeneration will continue to be explored further in the quest for brain repair.

## Author Contributions

DR, SG, and AH decided the layout, wrote the manuscript, acted Editors to this Research Topic, and selected the articles described therein. All authors contributed to the article and approved the submitted version.

## Conflict of Interest

The authors declare that the research was conducted in the absence of any commercial or financial relationships that could be construed as a potential conflict of interest.

